# P-146. Clinico-Demographic Profile and Drug Resistant Detection of Salmonella typhi Using Polymerase Chain Reaction and Blood Culture among Patient At San Lazaro Hospital, Philippines

**DOI:** 10.1093/ofid/ofae631.351

**Published:** 2025-01-29

**Authors:** Rolando D Quimpo, Edna m Edrada, Annavi Marie G Villanueva, Efren O Dimaano, Ruel O Teano

**Affiliations:** San Lazaro hospital, Manila, National Capital Region, Philippines; SAN LAZARO HOSPITAL, MANILA, National Capital Region, Philippines; San Lazaro hospital, Manila, National Capital Region, Philippines; San Lazaro hospital, Manila, National Capital Region, Philippines; San Lazaro hospital, Manila, National Capital Region, Philippines

## Abstract

**Background:**

In the last decade, multi-drug resistant (MDR) and extensively drug-resistant (XDR) microorganisms have been an emerging global health problem. The antimicrobial resistance surveillance of the Department of Health in 2019 reported 3 cases of resistant *Salmonella typhi* to ciprofloxacin. Due to the long turn-around time for blood cultures, management may be delayed for patients affected by MDR *S. typhi*. As such, there is a need to explore new methods for early detection of MDR *S. typhi.* The primary objective of this research is to determine the diagnostic application of Polymerase Chain Reaction (PCR) in early detection of MDR *S. typhi* among patients in the San Lazaro Hospital in the past 5 years.

**Table 1.**
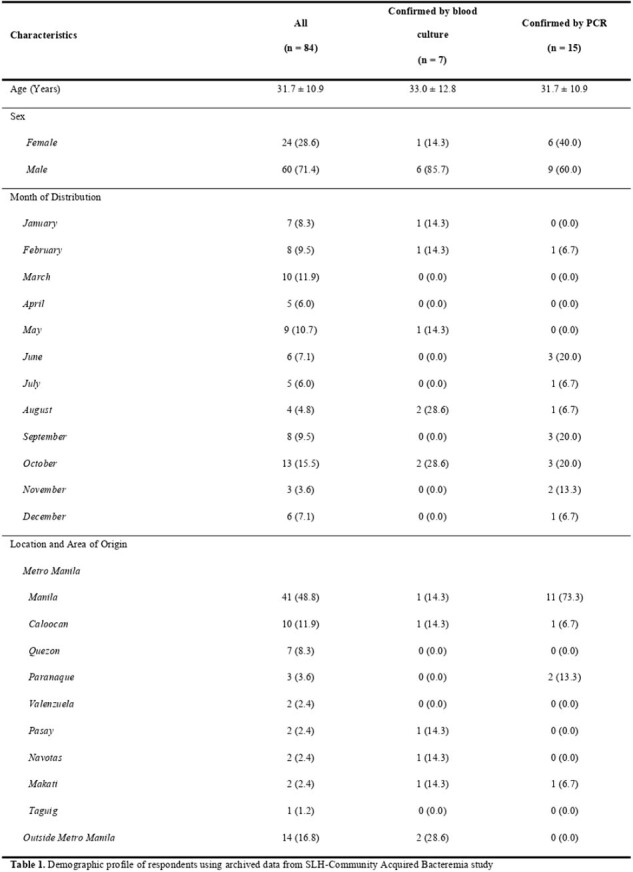
Demographic profile of respondents using archived data from SLH-Community Acquired Bacteremia study

**Methods:**

Clinical data were extracted from San Lazaro Hospital, PCR test results at SLH-Nagasaki Molecular Laboratory, Microbiology Laboratory and the medical records of San Lazaro Hospital. Data were analyzed using descriptive statistics, comparative analysis, and phi coefficient test.

**Table 2.**
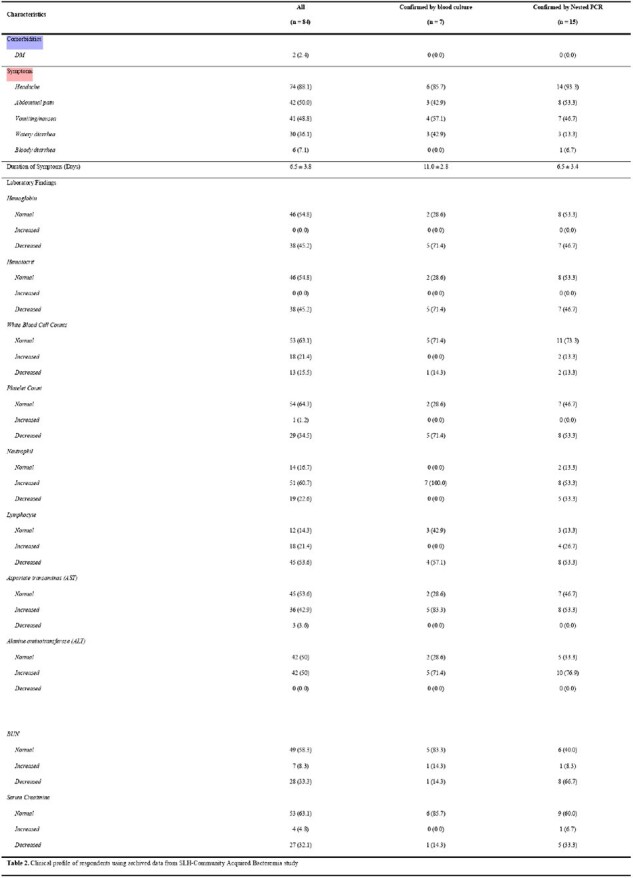
Clinical profile of respondents using archived data from SLH-Community Acquired Bacteremia study

**Results:**

Out of 84 admitted patients diagnosed of typhoid fever, seven (8.3%) were tested positive by blood culture while 15 (17.9%) were tested positive using Nested PCR method. Considering blood culture as standard, the Nested PCR method had sensitivity, specificity and accuracy of 14.29%, 81.82% and 76.19%; respectively, in detecting *S. typhi*. In addition, one sample from *gryA* gene group was tested resistant to Ciprofloxacin and two samples of *CatP* gene group were resistant to Chloramphenicol out of the 15 samples tested for AMR using Monoplex PCR.

**Table 3.**
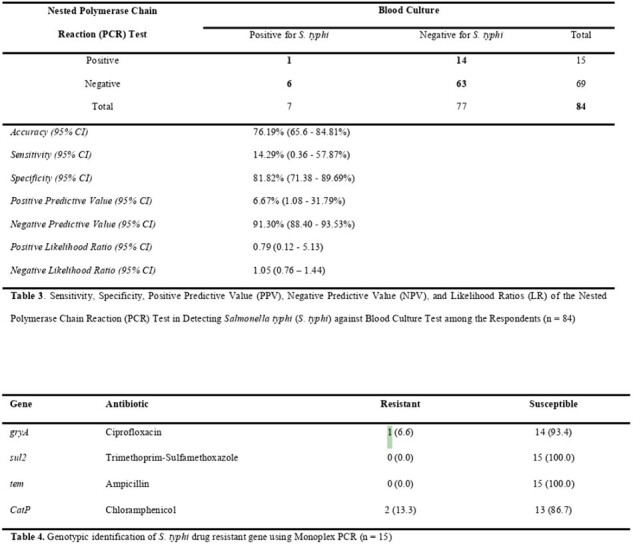
Sensitivity, Specificity, Positive Predictive Value (PPV), Negative Predictive Value (NPV), and Likelihood Ratios (LR) of the Nested Polymerase Chain Reaction (PCR) Test in Detecting Salmonella typhi (S. typhi) against Blood Culture Test among the Respondents (n = 84)Table 4. Genotypic identification of S. typhi drug resistant gene using Monoplex PCR (n = 15)

**Conclusion:**

The results of the PCR test and gene-specific PCR tests were not associated with those of the culture and sensitivity test. Due to the lack of MDR *S. typhi* cases detected via culture-sensitivity test necessary to examine the diagnostic capacity of PCR test, the ability of PCR in detecting MDR S. typhi remains unclear. Its utility as a reliable diagnostic test for the detection of MDR *S. typhi* requires further exploration and analyses.

Table 6.7.8. Sensitivity, Specificity, Positive Predictive Value (PPV), Negative Predictive Value (NPV), and Likelihood Ratios (LR) of the Monoplex Polymerase Chain Reaction (PCR-sul2) Test in Detecting Antibiotic Resistant Salmonella typhi (S. typhi) against Blood Culture Test Ciprofloxacin, Trimethoprime-Sulfamethoxazole and Ampicillin

**Figure:**
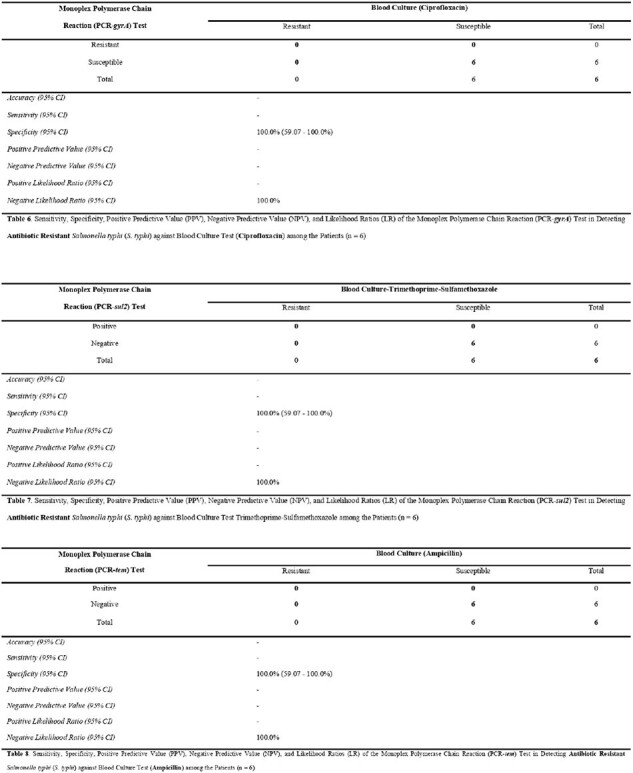

**Disclosures:**

**All Authors**: No reported disclosures

